# Enhanced tenogenic potential of tendon-derived mesenchymal stem cells: transcriptomic profiling and *in vivo* validation

**DOI:** 10.3389/fcell.2025.1687816

**Published:** 2025-10-16

**Authors:** Hadeer Khaled, Mohammed Zayed, Bumseok Kim, Byung-Hoon Jeong, Sang-Ik Oh

**Affiliations:** ^1^ Laboratory of Veterinary Pathology and Biosafety Research Institute, College of Veterinary Medicine, Jeonbuk National University, Iksan, Republic of Korea; ^2^ Korea Zoonosis Research Institute, Jeonbuk National University, Iksan, Republic of Korea; ^3^ Department of Bioactive Material Sciences, Jeonbuk National University, Jeonju, Republic of Korea; ^4^ Department of Surgery, College of Veterinary Medicine, South Valley University, Qena, Egypt

**Keywords:** mesenchymal stem cells, Achilles tendon, tendon-derived mesenchymal stem cells, bone marrow, RNA-seq, tendon repair

## Abstract

Tendon injuries represent a significant clinical challenge in the treatment of musculoskeletal disorders due to their restricted intrinsic regenerative capacity and propensity for scar tissue formation. Tendon-derived mesenchymal stem cells (TD-MSCs) are considered a promising therapeutic approach because of their ability to differentiate into tenocytes, modulate inflammation, and secrete trophic factors that facilitate tendon regeneration. However, the molecular mechanisms underlying their effects and therapeutic advantages over other MSC sources remain unclear. This study aimed to elucidate the molecular basis of the therapeutic potential of TD-MSCs through a comprehensive transcriptomic comparison with bone marrow-derived MSCs (BM-MSCs) and evaluate their tenogenic differentiation capacity and regenerative efficacy. We isolated and characterized TD-MSCs and BM-MSCs using flow cytometry, tri-lineage differentiation assays, and proliferation assays (CCK-8) and examined their transcriptomic profiles via RNA sequencing. Subsequently, the tenogenic differentiation potential of TD-MSCs was evaluated *in vitro* by analyzing the expression of key markers (*SCX*, *COL1*, *COL3*, *TN-C*, *TNMD*, *DCN*, *THBS-4*, and *SOX9*) using quantitative reverse transcription-polymerase chain reaction, along with protein levels of SCX, COL1, and COL3 via immunofluorescence. The therapeutic efficacy of TD-MSC treatment was further tested *in vivo* using a rat model of Achilles tendon injury, with histological and immunohistochemical analyses performed for 6 weeks post-injection. The results showed that TD-MSCs exhibited superior proliferation and a distinct transcriptomic profile, with significantly elevated expression levels of tenogenic genes (*COL1* and *TN-C*) compared to those observed in BM-MSCs. Following tenogenic induction, TD-MSCs showed enhanced differentiation capacity, with increased expression of tenogenic markers and downregulation of chondrogenic markers. *In vivo* treatment with TD-MSCs improved collagen fiber organization, enhanced structural integrity, and resulted in superior healing outcomes compared to untreated controls. These findings suggest that TD-MSCs possess intrinsic molecular advantages for tendon repair, characterized by enhanced tenogenic gene expression profiles relative to BM-MSCs and superior reparative potential both *in vitro* and *in vivo*. This study highlights the therapeutic potential of TD-MSCs for tendon regeneration and provides scientific evidence supporting tissue-specific MSC selection strategies for application in regenerative medicine.

## 1 Introduction

Tendon injuries represent a significant clinical challenge in musculoskeletal disorders (Leong et al., 2020; He et al., 2024), frequently leading to long-term disability and reduced quality of life. The natural healing process of tendons remains limited, often resulting in inferior mechanical properties that lead to chronic pain and functional impairment (Cottrell et al., 2016; He et al., 2024). Conventional therapeutic methods, including surgical intervention and rehabilitative therapy, typically achieve only partial restoration of native tendon structure and biomechanical function. Although these approaches may facilitate some degree of healing, the regenerated tissue generally lacks an organized extracellular matrix (ECM) and exhibits compromised mechanical strength compared with healthy tendons (Citro et al., 2025). These clinical limitations have driven the interest in regenerative medicine and cell-based therapies as promising options for improving tendon repair

Regenerative medicine approaches, particularly mesenchymal stem cell (MSC)-based therapies, have emerged as promising strategies for treating musculoskeletal disorders and tendinopathy (van den Boom et al., 2020; Mahmoud et al., 2022; Trapana et al., 2024). Many studies involving *in vivo* models have demonstrated that cell therapy effectively enhances tendon healing (Goldberg et al., 2024; Morya et al., 2024). MSCs demonstrate remarkable potential because of their ability to differentiate into tenocytes, modulate inflammation, and secrete trophic factors that support tissue repair (Costa-Almeida et al., 2019; Pittenger et al., 2019; Zayed et al., 2021; Citro et al., 2025). Importantly, MSCs from different anatomical sources retain a distinct molecular signature reflecting their developmental origins, a phenomenon termed “tissue memory” (Hass et al., 2011). Tissue-specific programming significantly influences regenerative potential via diverse gene expression profiles (Onizuka et al., 2020). Consequently, identifying a population of stem cells in the tendon tissue presents significant therapeutic potential for treating tendon injuries.

Tendon-derived MSCs (TD-MSCs) are clonogenic, multipotent, and capable of expressing both stem cell and tenogenic markers (Lui, 2015). TD-MSCs have been reported to have potential advantages over bone marrow-derived MSCs (BM-MSCs) for tendon repair, with TD-MSCs being more predisposed to tenogenic differentiation (Tan et al., 2012). At a molecular level, TD-MSCs are characterized by elevated expression of key tenogenic marker genes, including scleraxis (*SCX*), collagen type I (*COL1*), collagen type III (*COL3*), tenomodulin (*TNMD*), and tenascin-C (*TN-C*) (Tan et al., 2012; He et al., 2024). In addition, TD-MSCs exhibit stronger proliferation and tenogenic differentiation in co-culture with BM-MSCs (Wu et al., 2016). This inherent bias translates into a functionally greater capacity to form a tendon-like matrix *in vitro*. This superior molecular and functional profile enhances therapeutic outcomes *in vivo*. For instance, in a rat Achilles tendon rupture model, transplantation of TD-MSCs resulted in superior tendon repair compared to BM-MSCs, characterized by better-organized collagen fiber alignment, higher ultimate failure load, and increased COL1/COL3 expression (Al-Ani et al., 2015). These findings provide direct *in vivo* evidence that TD-MSCs can enhance tendon structure and biomechanical function, supporting their therapeutic relevance (Tan et al., 2012; Yea et al., 2023; He et al., 2024). However, the underlying molecular mechanisms driving this enhanced regenerative capacity remain poorly defined. Therefore, it is crucial to elucidate the molecular mechanisms via TD-MSCs that enhance their tenogenic potential for advancing clinical translation. Comprehensive transcriptomic analysis using RNA sequencing (RNA-seq) has emerged as a powerful approach for understanding stem cell biology and identifying the genes involved in specific biological processes (Wang et al., 2009). This technology enables the genome-wide characterization of gene expression patterns, providing an unbiased assessment of the molecular signatures that determine the regenerative therapeutic potential of MSCs.

We hypothesized that TD-MSCs would be naturally primed toward tendon regeneration and exhibit distinct gene expression signatures reflecting the enhanced activation of tenogenic regulatory pathways. To validate these predicted characteristics, we aimed to elucidate the molecular basis of the superior tenogenic potential of TD-MSCs by comprehensively comparing their transcriptome with that of BM-MSCs, a widely utilized MSC source for regenerative applications. We evaluated the therapeutic efficacy of TD-MSCs in a rat model of Achilles tendon injury. This integrated approach, which combines transcriptomic analysis with functional characterization, provides crucial mechanistic insights into specific tissue-derived stem cell therapies for tendon regeneration.

## 2 Materials and methods

### 2.1 Isolation and culture of MSCs

Sprague–Dawley pathogen-free seven-week-old male rats (*n* = 6, average weight 245 ± 16 g) were obtained from Samtako Co. (Samtako Bio, Korea) as MSC donors. The animals were maintained in a pathogen-free animal facility under standard laboratory conditions, with unrestricted access to food and water. All experimental procedures were approved by the Institutional Animal Care and Use Committee (IACUC) of Jeonbuk National University, Republic of Korea (NON2024-093).

The animals were euthanized via cervical dislocation. TD-MSCs were isolated and cultured as described previously (Ning et al., 2015). In brief, the Achilles tendon was completely dissected, and the peritendinous connective tissue and bone-tendon junction were removed. Tendons were placed in sterile phosphate-buffered saline (PBS), minced, and digested with collagenase type I (Fujifilm Wako Chemical, Japan) for 2 h at 37 °C. The medium was filtered through a 70-µm strainer and centrifuged (2,000 rpm, 5 min, 4 °C). The final cell pellet was pooled and resuspended in filtered Dulbecco’s modified Eagle’s medium (DMEM) supplemented with 10% fetal bovine serum and 1% antimycotic antibiotics and cultured at 37 °C in an atmosphere of 5% CO_2_. The cells were seeded at an initial density of 500 cells/cm^2^ in a 10 cm culture dish (Wu et al., 2020). Non-adherent cells were removed by changing the medium 48 h post-seeding. Subsequently, adherent cells were cultured to 80% confluence. Cells were trypsinized using 0.25% trypsin–EDTA and subcultured at a standard density of 5,000 cells/cm^2^ for expansion. BM-MSCs were obtained from the same rats, as described previously (Wu et al., 2023). In brief, the femurs were rinsed with PBS. Using an 18-gauge needle, bone marrow was extracted and transferred to a collection tube with media for flushing. Following centrifugation at 2,000 rpm for 5 min, the cell pellet was pooled, resuspended in media, and filtered twice through a 70-μm strainer. The TD-MSCs and BM-MSCs were cultured under the same conditions. The cells were expanded, cryopreserved in a Cell Banker 1 (Zenogen Pharma Co., Tokyo, Japan), and stored in liquid nitrogen for future use.

### 2.2 Cell proliferation and viability assay

The proliferative capacity of TD-MSCs and BM-MSCs was evaluated using a cell counting kit-8 colorimetric assay (CCK-8; Sigma-Aldrich, United States). Cells were seeded in 96-well plates at a density of 1 × 10^4^ cells per well (200 μL/well) and incubated for 24 h. Subsequently, 10 µL of CCK-8 reagent was added to each well in the dark, followed by 2 h of incubation at 37 °C with 5% CO_2_. The absorbance of the viable cells was measured at 450 nm using a microplate spectrometer (Molecular Devices, United States).

### 2.3 Tri-lineage differentiation of MSCs

The multipotent differentiation potential of TD-MSCs and BM-MSCs was confirmed using adipogenic, osteogenic, and chondrogenic lineage induction under specific *in vitro* conditions following a previously described protocol (Zayed et al., 2016). For adipogenic differentiation, confluent cells were treated with adipogenic induction medium containing DMEM supplemented with dexamethasone (1 μM), insulin (10 μg/mL), indomethacin (100 μM), and L-ascorbic acid (50 μg/mL) for 7 days. Lipid droplet accumulation was detected using Oil Red O staining. Osteogenic differentiation was induced in an osteogenic medium (DMEM with 50 µM ascorbic acid, 10 mM β-glycerophosphate, and 0.5 µM dexamethasone) for 14 days, and calcium deposition was confirmed using Alizarin Red staining. In chondrogenic differentiation, MSCs were cultured in DMEM supplemented with dexamethasone (100 nM), ascorbic acid (25 μg/mL), and TGF-β1 (10 ng/mL). Glycosaminoglycan deposition was assessed using Alcian Blue staining.

### 2.4 Immunophenotypic characterization

Flow cytometry analysis was performed according to the MSC criteria proposed by the International Society for Cellular Therapies to characterize the surface marker profile of TD-MSCs and BM-MSCs (CD90, CD45, and CD34). Cells were stained with fluorescein isothiocyanate (FITC)-conjugated antibodies. Mouse anti-rat CD90 (BD Pharmingen^TM^ FITC, Cat. #: 561,973, Korea), mouse anti-rat CD45 (BD Pharmingen^TM^ FITC, Cat. #: 561,867), and mouse anti-rat CD34 (BD Pharmingen^TM^ FITC, Cat. #: 560,238) were used. Isotype-matched IgG1 (BioLegend, Seoul, Korea) was used as a negative control. Fluorescence data were acquired using a NovoCyte® Flow Cytometer (NovoCyte 3000, ACEA Bioscience, Inc., United States), and data analysis was conducted using NovoExpress software (Agilent Technologies, United States).

### 2.5 Transcriptomic profiling and pathway analysis

RNA-seq was performed to compare the baseline transcriptomic profiles of TD-MSCs and BM-MSCs. Total RNA was isolated from passage 2 (*n* = 3 per group) using an RNeasy Mini Kit (QIAGEN, Hilden, Germany). Samples with an RNA integrity number (RIN) > 7, confirmed using an Agilent Bioanalyzer (Agilent Technologies, Santa Clara, CA, United States), and with yields of at least 0.5 µg were used for library preparation with the TruSeq Stranded Total RNA Kit (Illumina, San Diego, CA, United States). Paired-end sequencing (2 × 100 bp) was performed on an Illumina platform. Quality-filtered raw reads (Phred Q30) were aligned to the *Rattus norvegicus* reference genome (rn6) using HISAT2. Transcript abundance was quantified using StringTie software and normalized to the number of transcripts per million. Analysis of differentially expressed genes (DEGs) between TD-MSCs and BM-MSCs was performed using DESeq2, with significance criteria of a fold change ≥2 (|log_2FC| ≥ 1) and *p* < 0.05. Functional enrichment analysis of DEGs was performed using the g:Profiler for Gene Ontology (GO) terms, including biological process (BP), cellular component (CC), and molecular function (MF), with *p*-values adjusted using the Benjamini–Hochberg false discovery rate (FDR <0.05). Kyoto Encyclopedia of Genes and Genomes (KEGG) pathway enrichment (https://www.kegg.jp/kegg/pathway.html) was performed to identify significantly enriched signaling pathways ranked by adjusted *p*-values and gene ratios (proportion of DEGs within each pathway relative to total pathway genes).

### 2.6 Assessment of *in vitro* tenogenic differentiation

TD-MSCs were induced to undergo tenogenic differentiation according to a previously published study (Stanco et al., 2019). Cells were maintained in DMEM (Thermo Fisher Scientific) at 37 °C with 5% CO_2_ until 80%–90% confluence was reached. Then, the cells were cultured in a complete medium containing CTGF (100 ng/mL; PeproTech®, United States), ascorbic acid (50 μg/mL; Sigma-Aldrich, United States), and BMP-12 (50 ng/mL; BioLegend®, United States). The culture medium was refreshed every 3 days, and the progress in differentiation was monitored. Upon differentiation, quantitative reverse transcription polymerase chain reaction (qRT-PCR) and immunofluorescence staining were used to assess tenogenic marker expression at both the molecular and cellular levels.

### 2.7 RNA isolation and qRT-PCR

To evaluate the changes in gene expression during tenogenic differentiation, total RNA was isolated from undifferentiated and tenogenic-differentiated TD-MSCs using a GeneAll Biotechnology RNA Extraction Kit (Seoul, Korea). RNA concentration and purity were assessed using a NanoDrop 2000 Spectrophotometer (Thermo Fisher Scientific). Reverse transcription of mRNA into complementary DNA was performed using the ReverTra Ace® qPCR RT Master Mix with gDNA remover (Toyobo, Japan). qRT-PCR was performed on a CFX Opus 96^TM^ real-time PCR system (Bio-Rad Laboratories, United States) with SYBR Green Master Mix (Toyobo) to assess the expression of key tenogenic marker genes, including *SCX*, *COL1*, *COL3*, *TNMD*, *TN-C*, SOX9, thrombospondin 4 (*THBS-4*), and decorin (*DCN*), as described in Supplementary Table S1.

### 2.8 Immunofluorescence

TD-MSCs were cultured at a density of 2 × 10^4^ cells/well in a slide chamber (SPL Life Sciences). Following 7 days of tenogenic induction, the cells were fixed with 4% paraformaldehyde (PFA), washed with PBS, permeabilized using 0.1% Triton X-100 in PBS, and blocked using SuperBlock (ScyTek Laboratories, United States). Immunostaining was performed overnight at 4 °C with primary antibodies against SCX A-7 (mouse, 1:50; Santa Cruz Biotechnology, Dallas, TX, United States), COL1, and COL3 (1:200; Abcam, United Kingdom). After primary antibody binding, the cells were treated with Alexa Fluor 555-conjugated goat anti-mouse IgG for visualizing SCX and Alexa Fluor 488 goat anti-rabbit IgG for visualizing COL1 and COL3 (Invitrogen, United States) for 60 min at room temperature. The cells were mounted with an anti-fade mounting medium containing 4′,6-diamidino-2-phenylindole (DAPI) (Vector Laboratories, United States). Images were captured using a BX51 fluorescence microscope (Olympus, Tokyo, Japan). Fluorescence intensity was quantified using ImageJ software.

### 2.9 Rat model of Achilles tendon injury

**FIGURE 1:**
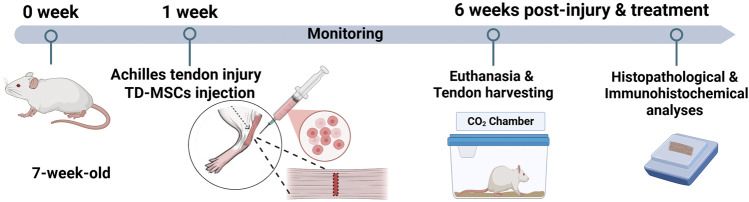
Schematic illustration showing the *in vivo* experimental design. An accommodation period of 1 week was allowed before the procedure, and TD-MSCs were injected at the same point after tendon defect creation and skin closure (day 0 of surgery). Rats were monitored for 6 weeks and then euthanized for tendon harvests to conduct histopathological and immunohistochemical analyses.

Sprague Dawley rats (*n* = 8) were randomly divided into two experimental injection groups (*n* = 4 each): control (untreated) and TD-MSCs (Figure 1). All rats were anesthetized via intramuscular injection of xylazine (10 mg/kg) and tiletamine/zolazepam (50 mg/kg). The animals were positioned in sternal recumbency, and the surgical area was shaved and disinfected. A small (approximately 1 cm) longitudinal skin incision was made on the right hind limb to expose the Achilles tendon. A standardized transverse transection defect was created at the midpoint of the tendon to preserve the paratenon using a digital micro-caliper with measurements in mm (Supplementary Figure S1; Zhang et al., 2024). The skin was sutured, and then 1 × 10^6^ TD-MSCs in 50 µL PBS were injected into the space between the Achilles tendon and the overlying skin. The control group received a PBS injection. To manage postoperative pain and prevent infection, the animals were administered ketorolac (3 mg/kg IM) and ampicillin (50 mg/kg IM) once daily for 3 days.

### 2.10 Histopathological evaluation and immunohistochemical analysis

All rats were euthanized 6 weeks post-injection as previously reported (Freedman et al., 2016; Freedman et al., 2017; Leahy et al., 2022). Achilles tendon tissues were collected from the injury site, immersed in 4% PFA for 48 h, and processed for paraffin embedding according to standard histological procedures. Tissue sections (5-μm thick) were prepared and stained with hematoxylin and eosin (H&E) for morphological examination. A semi-quantitative assessment was performed using a previously established scoring system (Yang et al., 2017). A score of 0 indicates normal tendon structure, while a score of 18 represents the most severe abnormality. Collagen architecture and organization were examined using Masson’s trichrome and picrosirius red staining, according to the manufacturer’s protocols.

Immunohistochemistry (IHC) was performed following the deparaffinization and rehydration of tissue sections. Antigen retrieval was achieved after incubating with proteinase K (120 mg, GeneAll Biotechnology Co., Korea) for 10 min at 37 °C. Nonspecific binding was prevented using SuperBlock (ScyTek Laboratories, United States), followed by overnight incubation at 4 °C with primary antibodies against SCX A-7 (Santa Cruz Biotechnology), COL1, and COL3 (Abcam, United Kingdom), each at a 1:200 dilution. Signal detection was accomplished by incubating for 1 h with a horseradish peroxidase-conjugated secondary antibody (Vector Laboratories) at room temperature. Visualization was performed using a Vectastain DAB Substrate Kit (Vector Laboratories) with hematoxylin counterstaining. Histological examination was conducted using a BX51 light microscope (Olympus Corp., Tokyo, Japan), and IHC staining intensity was quantified using FIJI (ImageJ) software.

### 2.11 Statistical analysis

All quantitative data were presented as the mean ± standard deviation (SD). Statistical comparisons between groups were performed using an unpaired Student’s t-test. Differences were considered statistically significant at ^*^ *p* < 0.05, ^**^ *p* < 0.01, and ^***^ *p* < 0.001. Data analysis was conducted using GraphPad Prism software version 8 (GraphPad Software Inc., United States).

## 3 Results

### 3.1 Isolation, characterization, and proliferation of TD-MSCs and BM-MSCs

**FIGURE 2:**
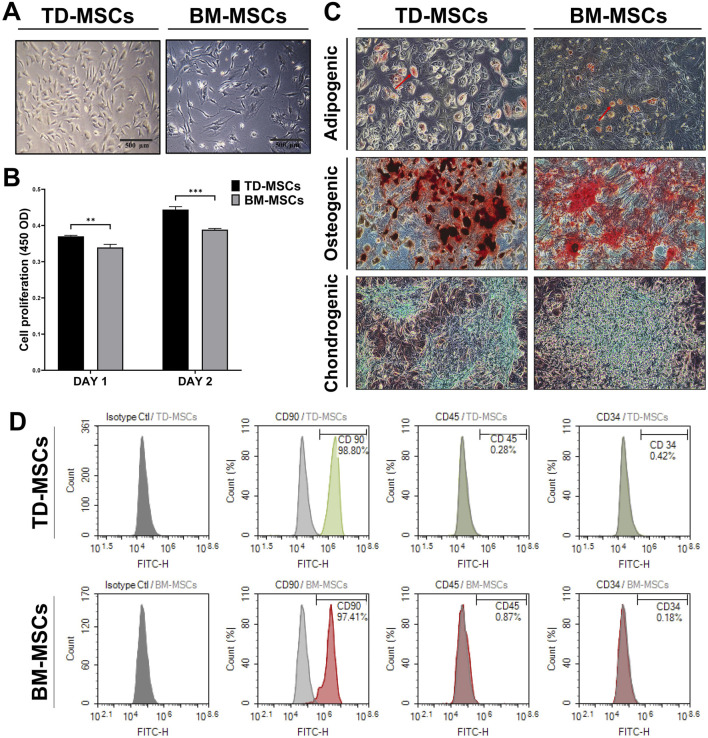
Comparative *in vitro* characterization of TD-MSCs and BM-MSCs. **(A)** Representative phase-contrast micrographs of TD-MSCs vs. BM-MSCs highlighting morphological differences (scale bars: 500 μm). **(B)** Cell proliferation rate on days 1 and 2. Data were presented as the mean ± SD (*n* = 3); **p < 0.01, and ***p < 0.001. **(C)** Tri-lineage differentiation potential of TD-MSCs and BM-MSCs (×100), confirmed using Oil Red O staining of lipid droplets for adipogenesis (red arrows), Alizarin Red staining of calcium deposits for osteogenesis, and Alcian Blue staining of glycosaminoglycan-rich matrix for chondrogenesis. **(D)** Flow cytometric analysis of surface markers. TD-MSCs and BM-MSCs showed >97% positive expression for CD90 and negative for CD45 and CD34 (with less than 1%).

BM-MSCs exhibited a typical spindle-shaped fibroblast-like morphology under an inverted phase-contrast microscope, whereas TD-MSCs were smaller and rounder (Figure 2A). Compared to BM-MSCs, quantitative analysis of cell proliferation on days 1 and 2 revealed a time-dependent increase in the number of TD-MSCs, with a significant increase observed on days 1 (*p* < 0.01) and 2 (*p* < 0.001) (Figure 2B). These results suggest that TD-MSCs possess an intrinsically greater *in vitro* proliferative capacity and regenerative potential than BM-MSCs. Both MSC populations demonstrated robust multipotency, differentiating into adipogenic, osteogenic, and chondrogenic lineages under the appropriate induction conditions (Figure 2C). Adipogenic-differentiated TD-MSCs and BM-MSCs showed Oil Red O-positive lipid droplets, indicating their ability to differentiate into adipocytes. Alizarin Red staining indicated mineralized matrix deposition (calcium nodules) in osteogenic cultures. Notably, TD-MSCs appeared to form more calcium nodules than BM-MSCs. During chondrogenic differentiation, cells were stained blue with Alcian Blue after induction, indicating the presence of a glycosaminoglycan-rich cartilage matrix. TD-MSCs and BM-MSCs were successfully isolated and characterized using flow cytometry (Figure 2D). The level of the MSC marker, CD90, was high in TD-MSCs (98.8%) and BM-MSCs (97.4%), while minimal expression of CD45 and CD34 was observed in TD-MSCs (0.3% and 0.4%, respectively) and BM-MSCs (0.2% and 0.9%, respectively). These findings confirmed the successful isolation of pure TD-MSCs and BM-MSCs.

### 3.2 Comparative transcriptomic profiling of TD-MSCs and BM-MSCs

To compare the baseline transcriptomes of TD-MSCs and BM-MSCs, we performed RNA-seq on undifferentiated cells from each source (*n* = 3 independent cell isolations; Supplementary Material 1). This analysis identified 2,654 DEGs between TD-MSCs and BM-MSCs (|log_2_ FC| ≥ 1, FDR <0.05). A clear separation was observed between the MSCs using unsupervised hierarchical clustering and heatmap analysis, suggesting distinct lineage-specific transcriptional profiles (Supplementary Figure S2A). Volcano plot analysis also highlighted the broad transcriptomic divergence between TD-MSCs and BM-MSCs (Supplementary Figure S2B), identifying 1,104 genes that were significantly upregulated and 1,550 genes that were downregulated in TD-MSCs relative to those in BM-MSCs.

**FIGURE 3:**
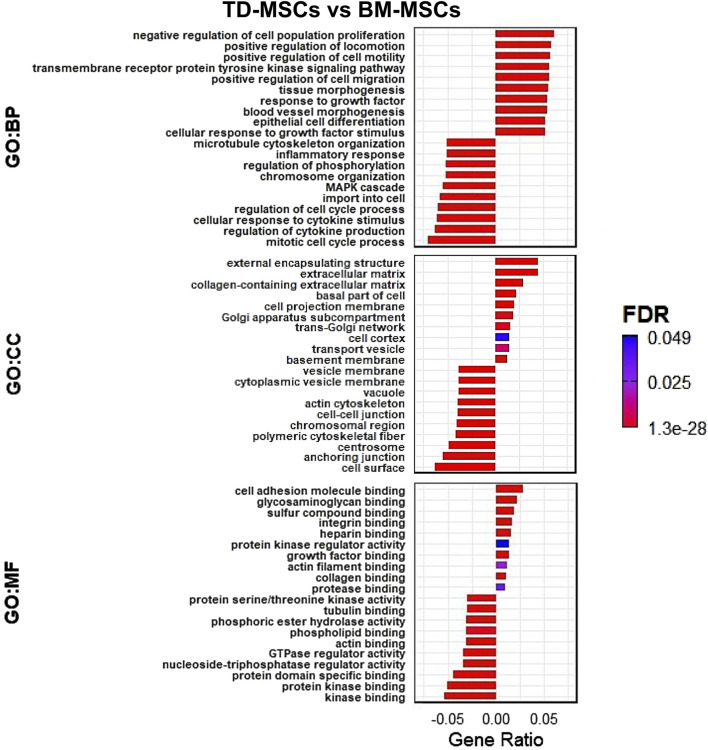
RNA-seq GO transcriptomic comparison of TD-MSCs and BM-MSCs. (A) GO enrichment analysis of DEGs, highlighting top enriched terms in biological processes (BPs), cellular components (CCs), and molecular functions (MFs) for TD-MSC vs. BM-MSC group comparison (bars represent the gene ratio, colored by FDR significance), *n* = 3 per group.

DEGs upregulated in TD-MSCs showed strong enrichment for BP of GO terms related to ECM organization and positive regulation of cell migration (Figure 3). Enriched GO terms in the CC category, including ECM and collagen-containing ECM, and the MF category, including integrin and growth factor binding, reflect the ECM adhesion and signaling roles of TD-MSCs in a regenerative environment.

**FIGURE 4:**
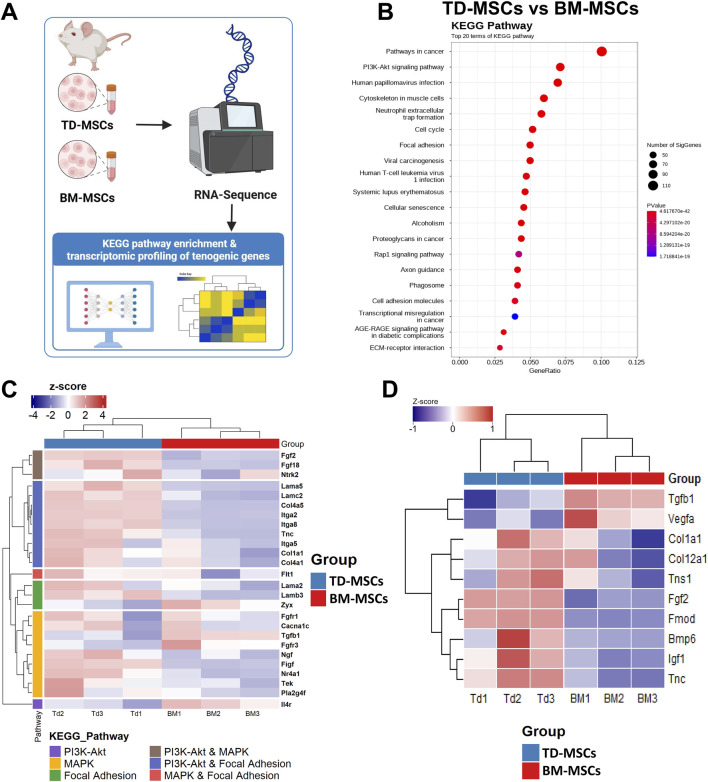
RNA-seq transcriptomic comparison of TD-MSCs and BM-MSCs. **(A)** Schematic illustration of the experimental workflow, pathway enrichment analysis, and tenogenic gene expression signatures. **(B)** KEGG pathway enrichment dot plot for DEGs in each pathway, where color indicates significance (FDR), *n* = 3 per group. **(C)** Heatmap of selected tenogenic-associated genes (normalized expression Z-scores) in BM-MSCs vs. TD-MSCs, focusing on genes in PI3K–AKT signaling, MAPK signaling, and focal adhesion pathways, *n* = 3 per group. **(D)** Heatmap of tenogenic-associated gene expression between BM-MSCs and TD-MSCs. The heatmap presents expression profiles of key tenogenic-associated genes across biological replicates, *n* = 3 per group.

The schematic workflow (Figure 4A) illustrates the transcriptomic profiling performed to compare TD-MSCs and BM-MSCs. KEGG pathway analysis showed that DEGs highly expressed in TD-MSCs were clustered in key regenerative pathways, such as PI3K–AKT signaling, MAPK signaling, focal adhesion, and ECM–receptor interaction (all FDR <0.001) (Figure 4B). These pathways are vital for cell proliferation, survival, ECM production, and differentiation during tissue repair. In contrast, the upregulated DEGs in BM-MSCs were associated with fewer pro-regenerative pathways; instead, the genes enriched in BM-MSCs included skeletal system development and hematopoietic cell lineage determination, indicating a difference in intrinsic biological predispositions.

### 3.3 Enhanced expression of tendon-specific genes in TD-MSCs compared to that in BM-MSCs

To evaluate the inherent ability of each MSC type for tenogenic differentiation, we performed a hypothesis-driven analysis of the tendon-related genes. The results revealed a significantly higher expression of key tendon ECM genes in TD-MSCs than in BM-MSCs (Figures 4C,D). TD-MSCs showed a marked upregulation of *FMOD* (507-fold) and *COL1A1* (2.3-fold), encoding the primary structural collagens in tendons (Zhang et al., 2019), along with *COL11A1* (4.9-fold) and *COL1A2* (3.2-fold). Furthermore, TD-MSCs exhibited notable increases in *ITGA2* (197.5-fold), *ITGA7* (6-fold), *ITGA8* (6-fold), and *BGN* (2.2-fold) expression. Significantly higher expression of genes encoding growth factors and cytokines such as *FGF18* (9-fold), *FGF2* (5.1-fold), *CTGF* (3.2-fold), *BMP2* (5.7-fold), and *BMP6* (5.3-fold) was also detected. Thus, the transcriptional profile of TD-MSCs was indicative of enhanced tendon ECM production and tenogenic signaling compared with BM-MSCs, which exhibited minimal expression of these genes. We further validated the strong tenogenic profile of TD-MSCs versus BM-MSCs based on the increased expression of tendon ECM synthesis and repair genes, such as *TN-C* (2.2-fold), encoding a glycoprotein critical for cell–ECM interactions during tendon healing (Xu et al., 2021). TD-MSCs also showed significant upregulation of *COL4A5* (25.9-fold), contributing to basement membrane formation, and MMP3 (68.4-fold), reflecting active ECM remodeling. The levels of cytokines, such as IL-33 (5.9-fold), were also significantly higher in TD-MSCs than in BM-MSCs. Additionally, TD-MSCs displayed specific upregulation of tenogenic-associated genes (*COL1A1*, *TN-C*, *FGF2*, and *FGF18*) (Figures 4C,D) within key KEGG pathways, including the PI3K–AKT, MAPK, and focal adhesion pathways. These pathways, which are enriched in TD-MSCs, are known for their essential roles in cell proliferation, differentiation, and ECM interactions necessary for tendon repair relative to BM-MSCs, implying potential differences in the tenogenic differentiation capacity of the MSC types.

### 3.4 Expression of tenogenic markers in TD-MSCs

**FIGURE 5:**
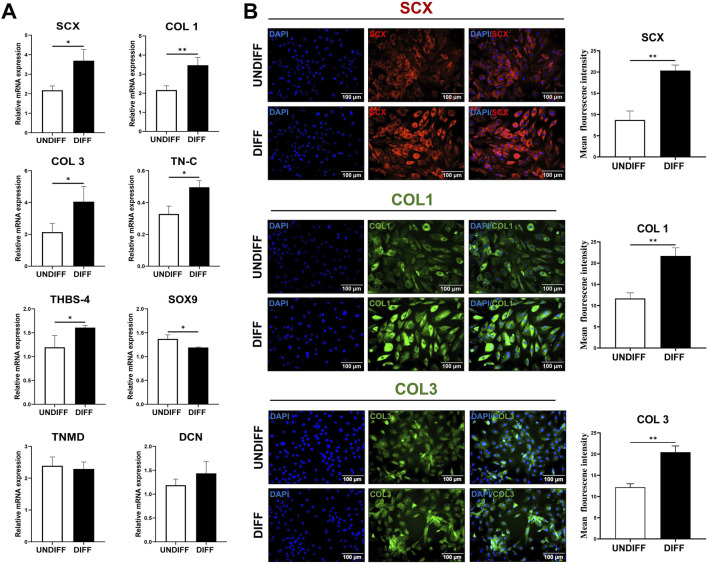
Tenogenic differentiation of TD-MSCs *in vitro*. **(A)** Relative mRNA expression of tenogenic marker genes in TD-MSCs under undifferentiated (UNDIFF) vs. differentiated (DIFF) conditions. Target genes included *SCX*, *COL1*, *COL3*, *TNMD*, *TN-C*, *SOX9*, *THBS-4*, and *DCN*. Data are presented as the mean ± SD (*n* = 3 per group); ^*^ *p* < 0.05 and ^**^ *p* < 0.01. **(B)** Representative immunofluorescence images of TD-MSCs before and after tenogenic induction of tenogenic markers. Cells were stained for SCX (red), COL1 (green), and COL3 (green) and with DAPI (blue) to visualize nuclei (×200, scale bar = 100 µm), *n* = 3 per group. Tenogenic-differentiated TD-MSCs showed stronger SCX nuclear localization and higher COL1/COL3 expression. Quantification of fluorescence intensity revealed that TD-MSCs exhibited a significant increase in tenogenic marker expression after differentiation (*n* = 3 per group, ^**^ *p* < 0.01).

Considering the superior baseline tenogenic transcriptomic profile of TD-MSCs compared with that of BM-MSCs, subsequent tenogenic differentiation analyses focused on TD-MSCs. Functional response to tenogenic differentiation was evaluated by determining the expression of key genes associated with tenogenic differentiation (Figure 5A). We hypothesized that baseline differences in RNA-seq might influence the *in vitro* differentiation potential. TD-MSCs responded strongly to tenogenic induction, with a significant upregulation of *SCX* (*p* < 0.05), *COL1* (*p* < 0.01), *COL3* (*p* < 0.05), *TN-C* (*p* < 0.05), and *THBS-4* (*p* < 0.05), whereas the expression of *DCN* and *TNMD* remained steady (*p* > 0.05) (Figure 5A), indicating their high tenogenic potential. During differentiation, TD-MSCs downregulated *SOX9* (*p* < 0.05) (Figure 5A), reflecting suppression of the chondrogenic pathway and a tendency to favor the tenogenic lineage. This was supported by the overexpression of tenogenic markers (*SCX*, *COL1*, *COL3*, and *TN-C*) (Figure 5A), highlighting a strong tenogenic response.

We measured the mean fluorescence intensity to evaluate the tenogenic capacity of tenogenic differentiation-protein markers. Representative images show immunostaining for SCX (red) and COL1 and COL3 (green) in undifferentiated and differentiated TD-MSCs. TD-MSCs showed a significant increase in SCX-positive nuclei (Figure 5B) and abundant COL1 and COL3 expression (Figure 5B). Quantitative analysis confirmed significantly higher fluorescence intensities in differentiated TD-MSCs, as evidenced by the increased expression of key tenogenic markers (*p* < 0.01) (Figure 5B).

### 3.5 *In vivo* tendon repair efficacy with TD-MSC treatment

**FIGURE 6:**
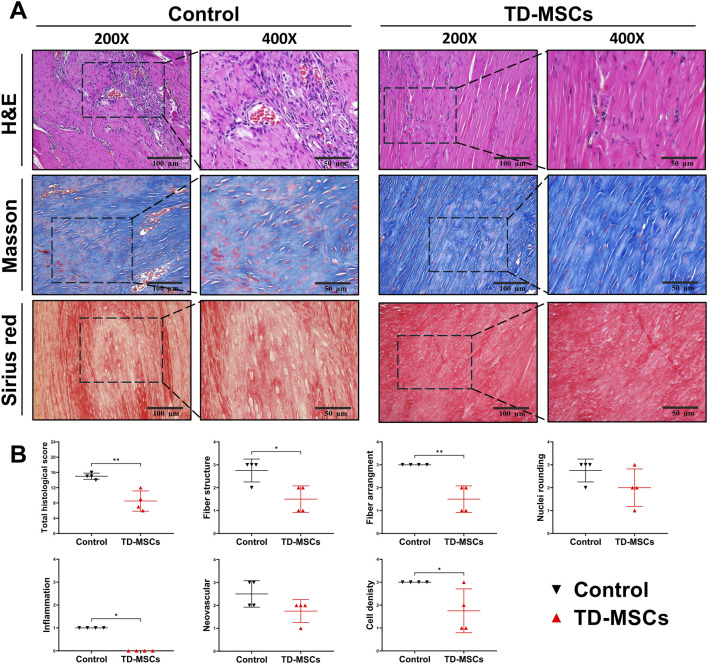
Outcomes of *in vivo* Achilles tendon healing at 6 weeks post-injury with TD-MSC treatment. **(A)** Representative histological images from untreated control vs. TD-MSC-treated tendon. Top: H&E staining illustrating overall tissue architecture, cellularity, and fiber alignment. Middle: Masson’s trichrome staining showed collagen deposition and alignment. Bottom: picrosirius red staining showed increased COL1 expression in the TD-MSC-treated group under brightfield (scale bars: 100 μm and 50 µm). **(B)** Semi-quantitative histological scoring of tendon healing based on H&E-stained sections; TD-MSC-treated tendons showed significantly lower histological scores than controls. Data are presented as the mean ± SD (*n* = 4 rats per group); ^*^ *p* < 0.05, and ^**^ *p* < 0.01.

**FIGURE 7:**
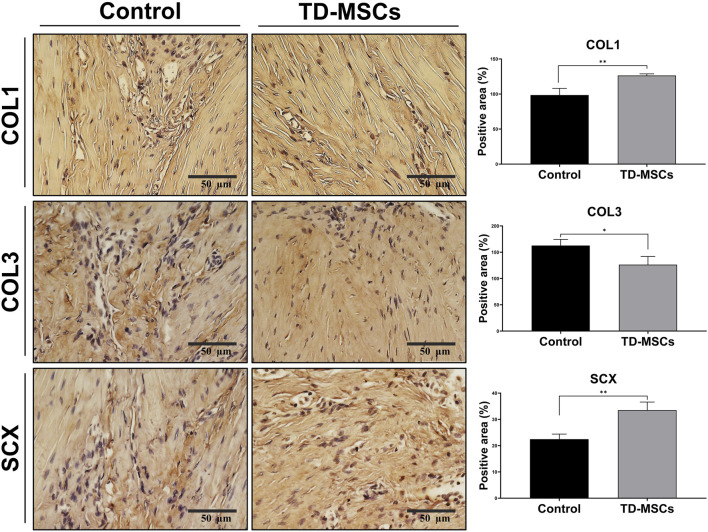
Immunohistochemical evaluation of tendon regeneration markers. Representative IHC images for COL1, COL3, and SCX expression from untreated control and TD-MSC-treated tendons (scale bar: 50 µm), with corresponding quantitative analysis. Data are presented as the mean ± SD (*n* = 4 animals/group; 3 fields/animal); ^*^ *p* < 0.05, and ^**^ *p* < 0.01.

Based on the superior profile and tenogenic differentiation capacity of TD-MSCs, we evaluated their therapeutic efficacy in a model of Achilles tendon injury. Rats received an injection of TD-MSCs (surgery day 0) around the Achilles tendon, while the untreated control received PBS only. H&E staining showed that the TD-MSC-treated tendons had a better-organized collagen fiber structure, alignment, and improved cellularity than the untreated control. The average total semi-quantitative histological scores of the TD-MSC treatment group (8.5 ± 2.65) were significantly lower than those of the control group (15 ± 0.82) (*p* < 0.01) (Figures 6A,B). Masson’s trichrome staining demonstrated increased collagen content and denser connective tissue in the TD-MSC-treated tendons, and picrosirius red staining showed stronger COL1 deposition in the treated tendons (Figure 6A). In IHC analysis, TD-MSC-treated tendons showed significantly higher expression of COL1 and SCX than control tendons (*p* < 0.01) and significantly lower expression of COL3 (*p* < 0.05) (Figure 7), reflecting a shift toward a more mature collagen matrix.

## 4 Discussion

Tendon tissue engineering has emerged as a promising field offering potential therapeutic strategies for repairing and regenerating damaged tendons and ligaments (Ning et al., 2023; Citro et al., 2025). MSCs derived from various tissues have been studied in tissue regeneration (Mahmoud et al., 2022; Margiana et al., 2022; Zayed et al., 2023; Trapana et al., 2024; Zhidu et al., 2024). Although MSCs share some fundamental characteristics, their tissue-specific characteristics can influence the therapeutic outcomes (Tan et al., 2012; Pizzute et al., 2015). TD-MSCs, which can differentiate into tenocytes, are at the forefront of research as the primary cellular source of tissue-engineered constructs (Zhang et al., 2023). This study evaluated the transcriptomic profiles of TD-MSCs and their potential for tenogenic differentiation and intralesional application *in vivo*. Considering that the bone marrow is the primary source of MSCs, we initially compared the characteristics of TD-MSCs with those of BM-MSCs according to the guidelines of the International Society for Cellular Therapy. Both cell types adhered to the culture dishes under standard culture conditions and expressed CD90 while lacking the expression of CD45 or CD34 (Rojewski et al., 2008). Although both MSC sources satisfy these standard criteria, BM-MSCs are known to exhibit cellular heterogeneity, and their yield decreases with larger aspiration volumes, which may limit their therapeutic reliability and scalability (Tan et al., 2012; Li et al., 2023). Therefore, TD-MSCs have been proposed as viable alternatives to BM-MSCs (Lui and Chan, 2011). Previous studies have suggested that compared to BM-MSCs, TD-MSCs could display enhanced colony-forming capacity, faster proliferation, and elevated expression of tenogenic markers and tendon-specific ECM components (Bi et al., 2007; Tempfer et al., 2009; Thaker and Sharma, 2012; Al-Ani et al., 2015). These biological characteristics may potentially facilitate tendon tissue repair. Our study supports this hypothesis as our findings indicate that TD-MSCs have a distinct tenogenic transcriptomic signature and exhibit enhanced tendon-specific differentiation and regenerative abilities *in vitro* and *in vivo*, supporting their potential utility as a cell source for tendon therapeutic applications.

RNA-seq analysis identified substantial baseline transcriptomic differences between TD-MSCs and BM-MSCs, with DEGs and pathway analyses emphasizing their distinct roles in tendon repair. TD-MSCs exhibited a notable difference, with significant enrichment in the PI3K–AKT, MAPK, and focal adhesion pathways, which are the key pathways involved in ECM remodeling, cell adhesion, proliferation, tenogenesis, collagen production, and tenocyte migration (Han et al., 2019; Zhang et al., 2020; Stańczak et al., 2024). Analysis of tenogenic-associated genes revealed elevated expression of key genes in TD-MSCs, including *TN-C*, *FGF18*, and *COL1A1*, suggesting enhanced ECM remodeling, growth factor signaling, and cell–matrix interactions. These findings indicated the inherent advantage of TD-MSCs in establishing a regenerative microenvironment conducive to tendon healing and enhanced tenogenic differentiation capacity. Notably, the PI3K–AKT pathway was enriched in TD-MSCs, which regulated *COL1* production and cell cycle progression during healing (Stańczak et al., 2024). *COL1A1* upregulation, crucial for ECM synthesis, has been observed in TD-MSCs (Tan et al., 2012; Yi et al., 2022). The activation of MAPK signaling in TD-MSCs corresponds to previous findings linking this pathway to FGF activity, collagen synthesis, ECM remodeling, and tenogenic differentiation of fibroblasts (Titan et al., 2019; Stańczak et al., 2024). The elevated expression of *FGF18* and *FGF2* in TD-MSCs further supports the involvement of this pathway in tenogenesis (Titan et al., 2019). Furthermore, this finding is consistent with the results of previous reports, indicating that growth factor pretreatment can promote tenogenic differentiation while minimizing ossification and that TD-MSCs intrinsically express tendon-related genes independent of external stimulation (Citro et al., 2025). Activation of the MAPK pathway also correlated with increased biglycan (BGN) expression, a critical small leucine-rich proteoglycan that regulates collagen fibrillogenesis (Tan et al., 2012; Zhang et al., 2019). In addition, focal adhesion pathway enrichment of integrins and related molecules, such as *ITGA2*, *ITGA8*, and *TN-C*, may improve cell–ECM adhesion and migration, allowing MSCs to respond more effectively to the biomechanical cues essential for tendon remodeling (Xu et al., 2021).

Following the identification of enhanced tenogenic gene expression in TD-MSCs compared to those in BM-MSCs using transcriptomic analysis, we focused our functional experiments on evaluating the differentiation capacity of TD-MSCs. The results confirmed the improved tenogenic differentiation of TD-MSCs *in vitro* and demonstrated their superior repair ability *in vivo* compared with untreated controls. Elevated expression of key tenogenic markers, including *COL1* and *TN-C*, suggests that TD-MSCs possess an inherent predisposition toward tendon regeneration. After tenogenic differentiation, TD-MSCs showed upregulation of genes, including *SCX*, *COL1*, *COL3*, *TN-C*, and *THBS-4*, compared to that observed in undifferentiated cells, reflecting their strong tendency to differentiate into tenocyte-like cells. SCX, a transcription factor specific to tendon progenitors, regulates *COL1* and *TNMD* and is essential for tendon development and maturation (Shukunami et al., 2006; Gaut and Duprez, 2016). COL1 is the main collagen in mature tenocytes, whereas COL3 supports early wound healing and formation of the epitenon and endotenon (Zhang et al., 2019). TN-C functions as a vital ECM protein that maintains tissue elasticity, especially during tendon injury and healing, when the biomechanical environment is disrupted (Pajala et al., 2009; Zhang et al., 2011; Al-Ani et al., 2015). TN-C is present in mature tendons, where it interacts with integrin receptors and other ECM components, influencing cell–matrix interactions and collagen fiber alignment (Li et al., 2021). Similarly, THBS-4 contributes to ECM organization by binding to various cellular receptors and ligands (Stenina-Adognravi and Plow, 2019). Although these proteins are primarily expressed in tenocytes, their expression also supports the ECM structure across different tissue types (Citro et al., 2025). This enhanced gene expression, validated using qRT-PCR and immunofluorescence, correlated with markedly elevated levels of SCX and collagen-related proteins in TD-MSCs. Moreover, TD-MSCs showed significant downregulation of *SOX9*, providing evidence that tenogenesis involves the suppression of chondrogenic pathways (Titan et al., 2019; Zhang et al., 2019).

Following the characterization of the TD-MSC population and confirmation of their tenogenic potential, we evaluated the therapeutic efficacy of TD-MSCs *in vivo* using a tendon injury model. Intratendinous injection of MSCs has become a widely used method for tendon repair in the treatment of musculoskeletal disorders, with promising clinical outcomes (Jo et al., 2018). To accurately assess the effects of TD-MSCs, all rats were euthanized, and their Achilles tendons were analyzed histopathologically and by IHC. H&E staining in the TD-MSC-treated group showed a higher degree of organized collagen fiber arrangement and reduced cellular disarray than in the controls. This improvement was reflected in the significantly lower histological scores, consistent with a previous study demonstrating that TD-MSCs treatment may facilitate tendon healing by promoting the formation of dense, longitudinally aligned collagen fibers and improving tissue organization (Al-Ani et al., 2015). These findings emphasize the importance of regulating collagen composition during tendon healing (Cui et al., 2011; Thankam et al., 2018). In contrast, untreated control tendons show elevated COL3 levels, which are typically associated with disorganized matrix and fibrotic remodeling (Tsai et al., 2006; Xu et al., 2018), whereas an optimal COL1/COL3 ratio is essential for functional tendon regeneration (Matsumoto et al., 2002; Zhang et al., 2020). A previous study has reported that TD-MSCs treatment contributed to connective tissue formation by continuously stimulating COL1 synthesis at the injury site, thereby supporting matrix maturation and mechanical strength (Al-Ani et al., 2015). Early upregulation of COL3 represents a normal part of the initial healing phase, providing temporary stabilization; however, its expression is expected to diminish as healing progresses. Reduced COL3 and increased COL1 levels suggest a shift toward tissue maturation, consistent with the natural remodeling process (Al-Ani et al., 2015). In this study, IHC analysis of the TD-MSC-treated group revealed increased expression of SCX and COL1 and reduced COL3 levels. These results suggest that TD-MSCs support regeneration by modulating the tendon microenvironment and promoting remodeling toward organized repair rather than fibrotic healing. Although previous studies have shown that TD-MSCs can differentiate into tenocytes and produce matrix components (Zhang et al., 2011; Shen et al., 2012; Thaker and Sharma, 2012; Al-Ani et al., 2015), we acknowledge that the present study did not provide direct evidence of transplanted cell tracking. Thus, we cannot assert that TD-MSCs themselves rapidly differentiated and served as the prime source of repair. Instead, our findings may support the therapeutic efficacy of local TD-MSC administration during the acute phase of injury, likely through their paracrine activity and immunomodulatory effects, which stimulated endogenous tendon cells and enhanced remodeling.

While our preclinical data highlight the therapeutic potential of TD-MSCs, translating these cells into clinical settings presents practical challenges. First, deriving MSCs from human tendons, usually obtained through biopsy or routine surgery (Perucca Orfei et al., 2021; Rizzo et al., 2025), is more invasive. However, this process could be made easier with minimally invasive, ultrasound-guided biopsy techniques (Murphy et al., 2013). Second, variability from donors remains a key factor for all cell-based therapies (Zayed and Iohara, 2023). For TD-MSCs, critical factors such as donor age, health status, and the source of the tendon biopsy significantly affect cellular properties (Yan et al., 2020). Future research should establish strict donor selection criteria to ensure consistent clinical outcomes, develop reliable *in vitro* potency tests to validate cell batches, and consider exploring allogeneic “off-the-shelf” cell banks from well-characterized, healthy donors.

This study provided important insights regarding the molecular basis of the transcriptional signature underlying the therapeutic potential of TD-MSCs for tendon regeneration. Our findings support the use of tissue-specific MSC sources in regenerative medicine. However, several limitations must be considered: the number and size of animals used for *in vivo* experiments were relatively small, healing outcomes were assessed at a single time point (6 weeks), biomechanical testing was not performed, which restricts the ability to correlate histological improvements with functional recovery, and the lack of evaluation of long-term therapeutic durability. In addition, cell tracking was not conducted, so we cannot determine whether injected TD-MSCs engrafted or primarily acted through paracrine mechanisms. Future studies should focus on scaling up TD-MSC production, evaluating their safety and efficacy in larger animal models, extending follow-up periods, and performing biomechanical assessments, including tensile strength and stiffness testing, to directly assess functional recovery.

## 5 Conclusion

Our findings demonstrate that TD-MSCs possess a distinctive transcriptomic profile characterized by significantly elevated expression of tenogenic genes, including *COL1* and *TN-C*, compared to BM-MSCs. *In vitro* experiments revealed that TD-MSCs showed robust tenogenic differentiation capacity compared to undifferentiated cells, as evidenced by the increased expression levels of *SCX*, *COL1*, *COL3*, *TN-C*, and *THBS-4*. *In vivo* evaluation showed that TD-MSC treatment improved tendon healing with enhanced collagen organization and structural integrity compared to untreated controls. Overall, our results highlight the tenogenic tendency of TD-MSCs and support their use as a promising cell source for tendon repair applications, warranting further preclinical and clinical investigations.

## Data Availability

All raw RNA-seq data were deposited in the NCBI Sequence Read Archive (SRA) under accession PRJNA1245624.

## Abbreviations

TD-MSCs, tendon-derived mesenchymal stem cells; BM-MSCs, bone marrow-derived mesenchymal stem cells; qRT-PCR, quantitative reverse transcription polymerase chain reaction; RNA-seq, RNA sequencing; DEGs, differentially expressed genes; GO, Gene Ontology; KEGG, Kyoto Encyclopedia of Genes and Genomes.
