# Supplementary material for: Enhanced tenogenic potential of tendon-derived mesenchymal stem cells: transcriptomic profiling and in vivo validation
Source: Front Cell Dev Biol. 2025 Oct 16;13:1687816. doi: 10.3389/fcell.2025.1687816 (PMC12574453; doi:10.3389/fcell.2025.1687816)
Supplement: Supplementary file 1 [file DataSheet2.docx]

**
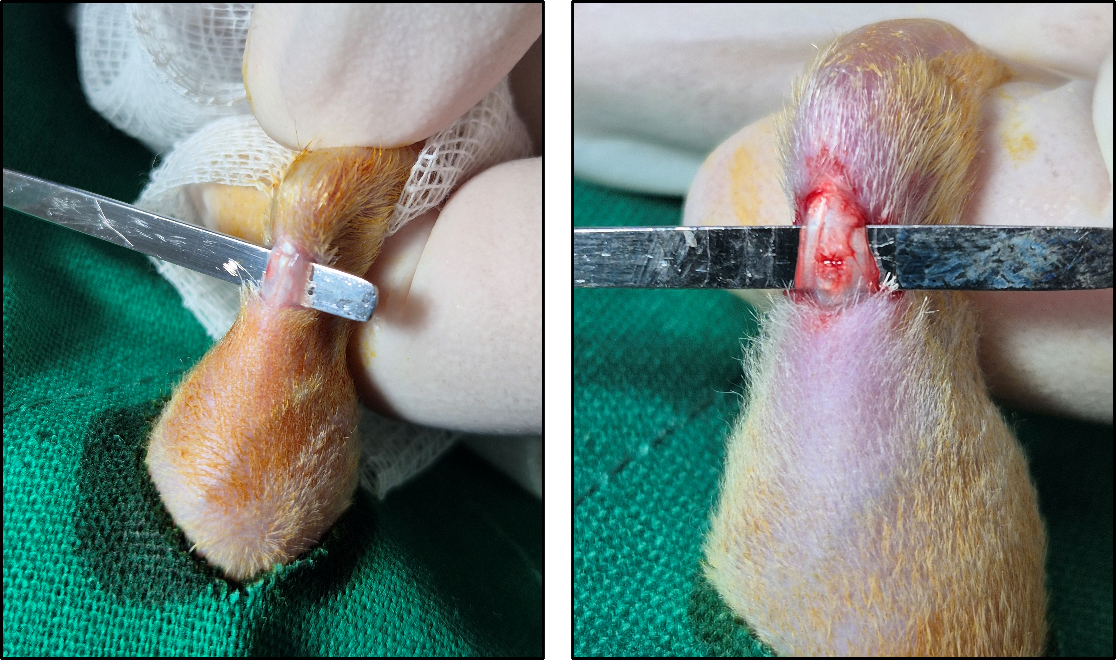
**

**Supplementary Figure 1. Intraoperative creation of a transverse defect in the Rat Achilles Tendon.** A standardized transverse transection defect was created in the mid-portion of the rat Achilles tendon using a scalpel (left, the Achilles tendon; right, transverse defect).


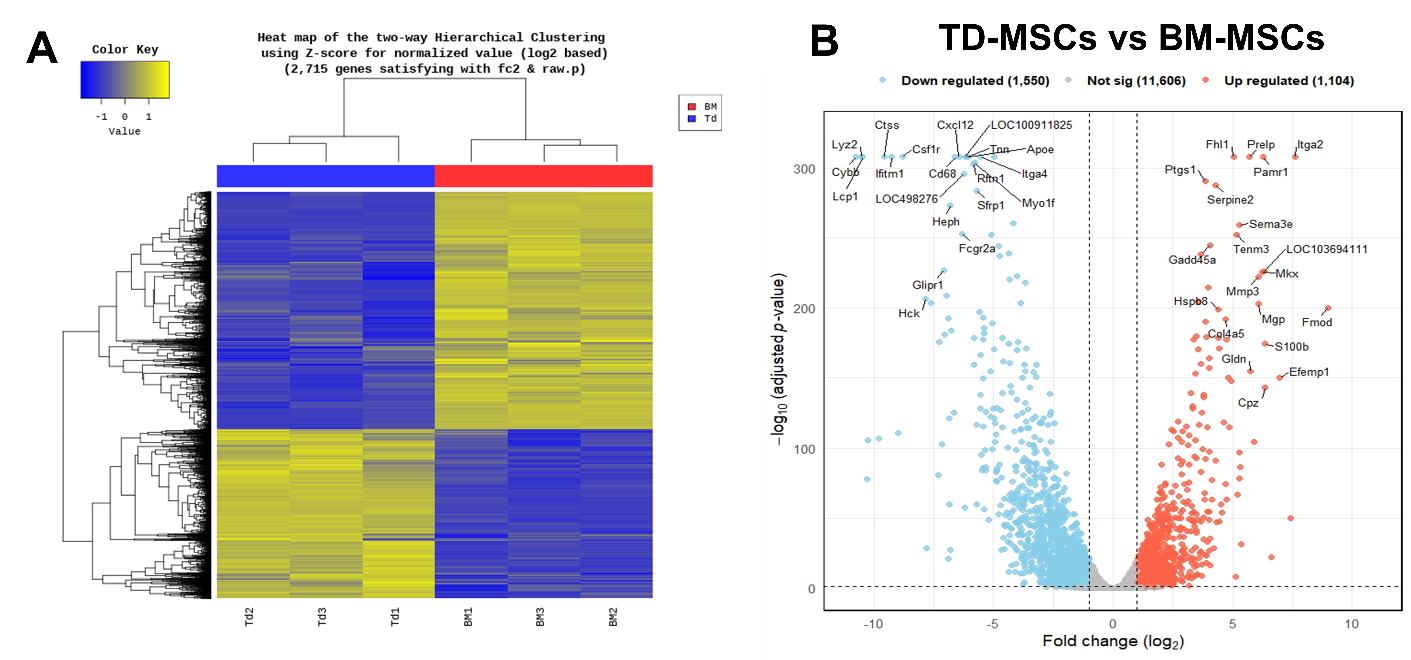


**Supplementary Figure 2. Transcriptomic profiling reveals distinct gene expression patterns in TD-MSCs and BM-MSCs.** (A) Heatmap of unsupervised hierarchical clustering for the top DEGs. (B) Volcano plot of gene expression differences between TD-MSCs and BM-MSCs.

**Supplementary Table 1. Real-Time PCR Primers Sequence (5' > 3') Information**

| Genes | Forward sequence | Reverse sequence |
| --- | --- | --- |
| *SCX* | TGGCCTCCAGCTACATTTCT | TGTCACGGTCTTTGCTCAAC |
| *COL1* | GCCAAGAAGACATCCCTGAA | GCAGAAAGGACAGCACTCGC |
| *COL3* | TGATGGGATCCAATGAGGGAGA | GAGTCTCATGGCCTTGCGTGTTT |
| *TN-C* | CAGAAGCCTTGGCCATGTG | GCACTCTCTCCCCTGTGTAGGA |
| *TNMD* | GGGATTGACCAGAATGAGCAA | GGTGCGGCGGGTCTTC |
| *SOX9* | GCACATCAAGACGGAGCAA | GGTTGTAGTGCGGAAGGTTG |
| *THBS-4* | AATACCATCCCTGCTACCC | TTCCGACACTCGTCAACA |
| *DCN* | TGGACTGAACCGTATGATTG | GATAGAAGTGGGCAGACCTT |
| *GAPDH* | TCTCTGCTCCTCCCTGTTCTA | ATGAAGGGGTCGTTGATGGC |
